# Probabilistic alternatives to Bayesianism: the case of explanationism

**DOI:** 10.3389/fpsyg.2015.00459

**Published:** 2015-04-27

**Authors:** Igor Douven, Jonah N. Schupbach

**Affiliations:** ^1^Sciences, Normes, Décision, Paris-Sorbonne UniversityParis, France; ^2^Department of Philosophy, University of UtahSalt Lake City, UT, USA

**Keywords:** Bayesianism, explanation, updating, inference, probability

## Abstract

There has been a probabilistic turn in contemporary cognitive science. Far and away, most of the work in this vein is Bayesian, at least in name. Coinciding with this development, philosophers have increasingly promoted Bayesianism as the best normative account of how humans ought to reason. In this paper, we make a push for exploring the probabilistic terrain outside of Bayesianism. Non-Bayesian, but still probabilistic, theories provide plausible competitors both to descriptive and normative Bayesian accounts. We argue for this general idea via recent work on explanationist models of updating, which are fundamentally probabilistic but assign a substantial, non-Bayesian role to explanatory considerations.

## 1. Introduction

There has been a probabilistic turn in the cognitive sciences, a development most prominently marked by the emergence of the “Bayesian paradigm” in the psychology of human learning and reasoning (e.g., Evans and Over, 2004; Griffiths and Tenenbaum, 2006; Tenenbaum et al., 2006; Gopnik and Tenenbaum, 2007; Oaksford and Chater, 2007, 2013; Over, 2009; Baratgin et al., 2013; Elqayam and Evans, 2013) and recent work on the “Bayesian brain” in cognitive neuroscience (e.g., Doya et al., 2006; Friston and Stephan, 2007; Hohwy, 2013). The vast majority of such work is—as in the examples cited above—described by adherents as “Bayesian.” In general, probabilistic and Bayesian approaches are so closely associated by cognitive scientists that it rarely is observed that these two approaches may come apart.

There are, nonetheless, various ways in which a theory might be probabilistic without being Bayesian. Most obviously, theories can draw upon probabilities interpreted in non-Bayesian ways (e.g., Gigerenzer and Hoffrage, 1995; Mayo, 1996; Williamson, 2010). But a theory can easily conflict with Bayesianism, even while adopting the standard Bayesian interpretation of probabilities (as measures of agent credences). In this paper, we want to highlight the potential merits of probabilistic, non-Bayesian accounts of this latter sort.

We focus our sights on the question of how humans update their confidences when confronted with new information^1^. Bayesian accounts model such updating strictly in accordance with

**Bayes's Rule**. Upon learning *A* ∈ 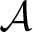 and nothing else between times *t*_1_ and *t*_2_, an agent's credences are to be updated so as to satisfy the equality Pr_*t*_2__(*B*) = Pr_*t*_1__(*B* | *A*) for all propositions *B* ∈ 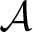, provided Pr_*t*_1__(*A*) > 0.

Here, 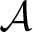 is an algebra of propositions over which the probability measures Pr_*t*_1__—representing the agent's credences at time *t*_1_—and Pr_*t*_2__—representing the agent's credences at later time *t*_2_—are defined, and Pr_*t*_1__(*B* | *A*) designates the prior (at *t*_1_) conditional probability of *B* given *A*.

The Bayesian account thus requires updates to be determined *purely* by an agent's prior conditional (subjective) probabilities. Probabilistic accounts more generally aim to model updating with the help of probability theory. Such accounts may accord with Bayes's Rule, but they need not. A non-Bayesian probabilistic account may, for example, calculate updated credences as a function of prior conditional probabilities plus some other set of factors (probabilistically explicable or not). In the following, we will be especially concerned with “explanationist” models of updating that take explanatory considerations into account in addition to prior conditional probabilities.

There are two crucially distinct ways one can interpret any theory of updating: as providing norms that updates rationally ought to satisfy, or as a descriptive model of how people in fact update. At the same time that cognitive scientists focusing on the descriptive interpretation have increasingly turned to probabilistic models, more and more philosophers have come to regard Bayesianism as providing the norms of both rational action and rational belief (e.g., Maher, 1993; Jeffrey, 2004; Joyce, 2009). Against this seemingly growing consensus on the nature of rationality, the present paper makes a push for exploring the probabilistic terrain outside of Bayesianism and challenges the thought that any deviation from Bayesianism implies a form of irrationality.

A central contention of this paper is that some probabilistic models of updating that conflict with Bayes's Rule constitute strong, plausible competitors to Bayes's Rule, whether the models in question are interpreted descriptively or normatively. We make a case for this claim by focusing on a particular family of non-Bayesian, probabilistic models of updating, namely explanationist models. We argue that explanationist models may be predictively more accurate than Bayesianism (Section 3) without being normatively defective in any way (Section 4). Probabilistic alternatives to Bayesianism accordingly deserve more explicit attention in cognitive science and philosophy than they have thus far received. Before making our case, however, in the next section we offer a general description of explanationism.

## 2. Explanationism

Deductive inference plays a key role in human reasoning. It is unsurprising, therefore, that this form of inference has been amply studied by psychologists (see, e.g., Evans, 1982; Evans and Over, 1996). Early on, psychologists commonly regarded deductive logic as providing standards of rational reasoning. But psychologists eventually came to realize that not all reasoning proceeds by deductive inference, and that the issue of rationality can arise also for forms of reasoning that are of a non-deductive nature. Having seen hundreds of white swans without ever having seen a swan of a different color, we may infer that all swans are white. While—as we now know—this inference would be to a false conclusion, it is not obviously irrational, and certainly more rational than if we inferred the same conclusion on the basis of having seen a mere handful of white swans, or after already having encountered a black swan. Indeed, many of our beliefs are seemingly held on the basis of this type of “inductive inference,” as it is now commonly called, and many of those beliefs would appear to be *rationally* held on that basis. So, it is again no surprise that there is a vast amount of work on this type of inference to be found in the psychological literature (see, e.g., Rips, 2001; Heit and Feeney, 2005; Heit, 2007; Heit and Rotello, 2010).

What *is* surprising is the almost complete neglect by psychologists of a form of inference that is neither deductive nor inductive but that does seem to play a key role—for better or worse—in human thinking. The form of inference we mean has been labeled “abductive inference” (or “abduction”) by the great American pragmatist philosopher Charles Sanders Peirce. (See the supplement on Peirce of Douven, 2011 for references). Abduction and induction distinguish themselves from deduction by being *ampliative*: unlike deductively valid inferences, cogent abductive and inductive arguments do not guarantee the truth of a conclusion on the basis of the truth of the premises. Abduction then distinguishes itself from induction by giving pride of place to explanatory considerations, in that it makes the believability of a hypothesis partly a matter of how well the hypothesis explains the available evidence.

To illustrate, consider the following famous anecdote about the invasion of the Thames by the Dutch fleet in 1667—also known as “the Raid on the Medway”—and Sir Isaac Newton, who was a Fellow at Trinity College, Cambridge, at the time:

Their guns were heard as far as Cambridg, and the cause was well-known; but the event was only cognizable to Sir Isaac's sagacity, who boldly pronounc'd that they had beaten us. The news soon confirm'd it, and the curious would not be easy whilst Sir Isaac satisfy'd them of the mode of his intelligence, which was this; by carefully attending to the sound, he found it grew louder and louder, consequently came nearer; from whence he rightly infer'd that the Dutch were victors. [William Stukeley, *Memoirs of Sir Isaac Newton's Life*, quoted in Westfall (1980 p. 194)]

The “mode of intelligence” referred to here, which according to Westfall (1980, p. 194) struck the other Fellows in Cambridge with awe, is most plausibly thought of as involving abductive reasoning. It is exceedingly difficult to think of a reasonable set of premises—reasonable from Newton's perspective at the time—from which the conclusion that the Dutch had won follows deductively. Nor did the Dutch—or any other nation that possessed a sizable fleet in the second half of the seventeenth century—invade England frequently enough for Newton's reasoning to be naturally construed as inductive. Rather, it seems that what led Newton to his conclusion is that a Dutch victory was the *best explanation* for his evidence: there are various potential explanations of why the sound of the canon fire grew louder and louder that do not involve a Dutch victory. For instance, the British fleet might have defeated the Dutch, but then that victory might have been followed by a mutiny in which the British marines turned against their own headquarters. However, this and other alternative potential explanations are topped, in terms of explanatory goodness, by the hypothesis that the Dutch fleet had beaten the British.

Abduction has been identified as playing a central role in scientific reasoning by various historians and philosophers of science (e.g., McMullin, 1984, 1992; Lipton, 1993, 2004; Achinstein, 2001). McMullin (1992) even refers to abduction as “the inference that makes science.” This is not to say that abduction has no place outside of science. Various authors have argued for its prominence in everyday contexts as well, for instance, that abductive reasoning is routinely and automatically invoked when we rely on the words of others (Harman, 1965; Adler, 1994; Fricker, 1994) and even in interpreting the words of others (e.g., Bach and Harnish, 1979, p. 92; Hobbs, 2004). In philosophy, abduction has been relied on in defenses of the position of scientific realism, according to which science progressively succeeds in providing better and better representations of reality (Boyd, 1984; Psillos, 1999), as well as in defenses of various metaphysical theses (e.g., Shalkowski, 2010).

A more modern name for abduction is “Inference to the Best Explanation” (IBE), and most statements of abduction to be found in the literature are rather straightforward unpackings of that name. In Musgrave's (1988, p. 239) formulation, for instance, abduction is the principle according to which “[i]t is reasonable to accept a satisfactory explanation of any fact, which is the best available explanation of that fact, as true,” and Psillos (2004, p. 83) tells us that “IBE authorizes the acceptance of a hypothesis *H*, on the basis that it is the best explanation of the evidence.” Such formulations raise questions of their own. What makes one explanation better than others? When is an explanation satisfactory? And, ought we really to accept the best explanation of the evidence even if it explains the evidence very poorly? Moreover, one wonders what the relationship between abduction and Bayesianism might be, given that abduction is apparently stated in terms of the categorical notion of acceptance, and does not refer to probabilities or credences.

In recent years, researchers have become interested in a version of abduction that is probabilistic in nature and even has Bayes's Rule as a limiting case (Douven, 2013; Douven and Wenmackers, in press). Where {*H_i_*}_*i*≤*n*_ is a set of self-consistent, mutually exclusive, and jointly exhaustive hypotheses, this version of abduction models human learning as an act of updating one's degrees of belief on new evidence in accordance with

**Probabilistic abduction**. Upon learning *E* ∈ 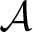 and nothing else between times *t*_1_ and *t*_2_, an agent's credences are to be updated so as to satisfy the equality
  (1) $${\mathrm{Pr}}_{t_{2}}(H_{i})\;=\;\frac{{\mathrm{Pr}}_{t_{1}}(H_{i}){\mathrm{Pr}}_{t_{1}}(E|H_{i})+ℰ(H_{i},E)}{\sum_{j\;=\;1}^{n}{\mathrm{Pr}}_{t_{1}}(H_{j}){\mathrm{Pr}}_{t_{1}}(E|H_{j})+ℰ(H_{j},E))},$$
  with ℰ assigning a bonus to the hypothesis that explains the evidence best, and nothing to the other hypotheses, and supposing Pr_*t*_1__(*E*) > 0.

It is easy to verify that probabilistic abduction concurs with Bayes's Rule if ℰ is set to be the constant function 0, meaning that no bonus points for explanatory bestness are ever attributed. It is not much more difficult to verify that probabilistic abduction concurs with Bayes's Rule *only if* no bonus points are assigned (Douven and Wenmackers, in press).

Naturally, as stated here, probabilistic abduction is really only a schema as long as ℰ has not been specified. For present purposes, this matter can be left to the side. In fact, for this paper, the rule only serves to show that there are versions of abduction that are direct contenders to Bayes's Rule. But one can think of many more probabilistic update rules that explicate the broad idea that explanatory considerations have confirmation-theoretic import—the central idea underlying abduction. Rather than advocating any particular such rule, we now proceed to argue that the Bayesian model of updating—whether construed descriptively or normatively—may plausibly be improved in various ways by taking into account explanatory considerations, leaving the details of how exactly to account for such considerations for another occasion.

## 3. Explanationism vs. bayesianism: descriptive adequacy

Contrary to what the growing popularity of Bayesianism among psychologists might lead one to expect, studies regularly find that people update in ways inconsistent with the Bayesian model; see, for instance, Phillips and Edwards (1966), Robinson and Hastie (1985), and Zhao et al. (2012)^2^. What is more, there is evidence suggesting that explanatory considerations do have an impact on people's beliefs; see, for instance, Koehler (1991); Pennington and Hastie (1992); Josephson and Josephson (1994); Thagard (2000); Lombrozo (2006, 2007, 2012); Lombrozo and Carey (2006); Douven and Verbrugge (2010); Bonawitz and Lombrozo (2012); Legare and Lombrozo (2014), and Lombrozo and Gwynne (2014).

The typical reaction to such findings is to look on departures from Bayesian reasoning as a complication or problem, and subsequently to hunt for explanations for why people are ostensibly straying from the proper rational norms. A far less explored option is to question whether Bayes's Rule (and with it Bayesianism) describes the appropriate normative standard for updating. We ask the normative question in the next section. In this section, we explore whether probabilistic models that take into account explanatory considerations might do better at describing people's updating behavior than Bayes's Rule.

The non-Bayesian, probabilistic models that we examine are related to research reported in Douven and Schupbach (in press), which in turn built on research reported in Schupbach (2011). The focus of the latter paper was on probabilistic measures of explanatory goodness or “power,” which aim to formalize the degree to which a potential explanation *H* accounts for evidence *E*. For example, according to a very simple proposal, *H* explains *E* to a degree equal to Pr(*E* | *H*) − Pr(*E*). Other—prima facie more promising—measures that have been discussed in the philosophy of science literature include Popper's (1959) measure,

(2) $$\frac{\mathrm{Pr}(E|H)-\mathrm{Pr}(E)}{\mathrm{Pr}(E|H)+\mathrm{Pr}(E)},$$

Good's (1960) measure,

(3) $$\ln\left(\frac{\mathrm{Pr}(E|H)}{\mathrm{Pr}(E)}\right)\text{​},$$

and Schupbach and Sprenger's (2011) measure,

(4) $$\frac{\mathrm{Pr}(H|E)-\mathrm{Pr}(H|\neg E)}{\mathrm{Pr}(H|E)+\mathrm{Pr}(H|\neg E)}.$$

It is to be noticed that, while all three measures have 0 as the “neutral point,” they are not all on the same scale. In particular, Popper's and Schupbach and Sprenger's measures have range [−1, 1] while Good's measure has range (−∞, ∞). However, Schupbach (2011) also considers functional rescalings of Good's measure obtained via this schema:

(5) $$L_{\alpha }(x)\;=\;\{\begin{array}{cc} 1-e^{-x^{2}2\alpha^{2}} & \text{if }x\;⩾\text{0};\\ -1+e^{-x^{2}2\alpha^{2}} & \text{if }x<\text{0}, \end{array}$$

which do all have range [−1, 1]. Below, we use “*L_a_*” to refer to the rescaling of Good's measure obtained in this way with α = *a*.

Schupbach (2011) sought to answer the question of how well these and some other measures of explanatory goodness capture people's judgments of explanatory goodness. To that end, an experiment was conducted in which 26 participants were individually interviewed. In the interviews, the participants were shown two urns containing 40 balls each, with one urn (“urn A”) containing 30 black balls and 10 white ones, and the other urn (“urn B”) containing 15 black balls and 25 white ones. Each interview started by informing the participant about the contents of the urn and giving him or her a visual representation of these contents—which remained in sight during the whole interview. The experimenter then tossed a fair coin and decided, based on the outcome, whether urn A or urn B would be chosen. The participant knew that an urn was chosen in this way, but was not informed about which urn had been selected. Instead, the experimenter drew 10 balls from the selected urn, without replacement, and lined up the drawn balls in front of the participant. After each draw, participants were asked: (i) to judge the explanatory goodness, in light of the draws so far, of the hypothesis that urn A had been selected (*H*_A_); (ii) to do the same for the hypothesis that urn B had been selected (*H*_B_); and (iii) to assess how likely it was in the participant's judgment that urn A had been selected, given the outcomes at that point. The participant had to answer the questions about explanatory goodness by making a mark on a continuous scale with five labels at equal distances, the leftmost label reading that the hypothesis at issue was an extremely poor explanation of the evidence so far, the rightmost reading that the hypothesis was an extremely good explanation, and the labels in between reading that the hypothesis was a poor/neither poor nor good/good explanation, in the obvious order.

The data obtained in this experiment allowed Schupbach to calculate, for each participant and for each of the measures that he considered, the explanatory power of *H*_A_ and *H*_B_ after each draw the participant had witnessed, where either objective probabilities or credences could be used for the calculations. The results of these calculations were compared with the actual judgments of explanatory goodness that the participant had given after each draw. The results somewhat favored Schupbach and Sprenger's (2011) measure over its competitors. In general, however, Popper's measure, various rescalings of Good's measure, and Schupbach and Sprenger's measure all performed well in predicting participant judgments concerning explanatory power—regardless of whether explanatory power was calculated on the basis of objective probabilities or on the basis of credences.

In Douven and Schupbach (in press), the data gathered in Schupbach's experiment were re-analyzed for a very different purpose. Whereas Schupbach used credences as well as objective probabilities to calculate values of explanatory goodness according to the above measures, which were then compared with participants' judgments of explanatory goodness, Douven and Schupbach were instead interested in the role that such judgments play in updating credences. Put differently, where Schupbach took judgments of explanatory goodness to be the response variable and either credences or objective probabilities as the input for one of the measures of explanatory goodness, the output of which then served as the predictor variable, Douven and Schupbach took credences as the response variable and objective probabilities and judgments of explanatory goodness as possible predictors. In doing so, they hoped to shed light on the question of the role of explanatory considerations in updating, in particular, of whether taking into account such considerations, possibly in conjunction with objective probabilities, leads to better predictions of people's updates—as should be the case, according to the descriptive reading of explanationism.

To be more precise, Douven and Schupbach (in press) first collected the credences of all participants into one variable (call this variable “S”), the objective conditional probabilities that those credences should have matched for the updates on the draws to obey Bayes's Rule into a second variable (call this “O”), the judgments of explanatory goodness of *H*_A_ into a third variable (“A”), and the judgments of explanatory goodness of *H*_B_ into a fourth (“B”). They then fitted a number of linear regression models, with S as response variable and with all or some of O, A, and B as predictor variables. The most interesting comparison was between the Bayesian model (called “MO” in the paper), which had only O as a predictor variable, and the full, explanationist model (“MOAB”), which had O, A, and B as predictor variables. In this comparison, as in the general comparison between all models that had been fitted, the explanationist model clearly came out on top. The difference in AIC value between MO and MOAB was over 120 in favor of the latter. Also, MOAB had an *R*^2^ value of 0.90, while MO had an *R*^2^ value of 0.83. A likelihood ratio test also favored MOAB over MO: χ^2^_(2)_ = 124.87, *p* < 0.0001.

In short, the explanationist model MOAB was much more accurate in predicting people's updates than the Bayesian model MO, strongly suggesting that, at least in certain contexts, agents's explanatory judgments play a significant role in influencing how they update. Note that, by accepting this conclusion, one is not leaving the probabilistic paradigm: conditional probabilities figure as a highly significant predictor in MOAB as well. The conclusion *is* strongly non-Bayesian, however, insofar as MOAB identifies explanatory judgments as significant predictors, too, in conflict with what ought to hold if people were strict Bayesian updaters.

The previous research showed that, in a context in which one is trying to predict people's updated credences, if next to objective probabilities one has access to people's explanatory judgments, one is well-advised also to take the latter into account. In reality, however, we rarely know people's explanatory judgments. Does explanationism suggest anything helpful in contexts in which only objective probabilities are available? It may well do so. Provided we have all the probabilistic information at hand that is required as input for the measures of explanatory power stated above, we can use the output of those measures in combination with objective probabilities and try to predict someone's updates on that combined basis. Given that Schupbach (2011) found a number of the measures of explanatory power to capture well people's judgments of explanatory power, and given that Douven and Schupbach (in press) found people's judgments of explanatory power to co-determine significantly their subjective probabilities, there is reason to believe that objective probabilistic information alone allows one to improve upon Bayesian models, which ignore explanatory considerations altogether.

In Douven and Schupbach (in press), only judgments of explanatory goodness were taken into account; no degrees of explanatory goodness determined by any measure of explanatory power were considered. To see whether such degrees of explanatory goodness (derived from the objective probabilistic information available) help make more accurate predictions about people's updates, we had another look at the data from Schupbach (2011) and fitted a series of linear models similar to MOAB, but now with participants's judgments of explanatory goodness replaced with calculated degrees of explanatory goodness. Specifically, we constructed linear models with S as response variable and O, degrees of explanatory goodness of *H*_A_, and degrees of explanatory goodness of *H*_B_ as predictors. Values of the last two predictors were determined in five distinct ways: using Popper's measure, using three separate rescalings of Good's measure (*L*_0.5_, *L*_1_, *L*_2_), and using Schupbach and Sprenger's measure. In the following, variable “Y_X_” represents degrees of explanatory goodness for hypothesis *H*_Y_ (Y ∈ {A, B}) calculated using measure X ∈ {P, G1, G2, G3, SS}, where “P” stands for Popper's measure, “G1” for *L*_0.5_, which is the first rescaled version of Good's measure, and so on. Similarly, “MXYZ” names the model with predictors X, Y, and Z.

Table 1 gives some important statistics for comparing the models, where we have also included MO from Douven and Schupbach (in press). Because MO is nested within each of the other models, it could be compared with them by means of likelihood tests. The χ^2^ column in Table 1 gives the outcomes of these tests, which were all in favor of the richer model. Given that the χ^2^ values obtained in the tests were all significant, this is a first indication that any of the explanationist models provides a better fit with the data than the Bayesian model. Naturally, the better fit might be due precisely to the fact that the explanationist models include more predictors than MO. For that reason, it is worth looking also at the AIC metric, which weighs model fit and model complexity against each other and penalizes for additional parameters. Burnham and Anderson (2002, p. 70) argue that a difference in AIC value greater than 10 indicates that the model with the higher value enjoys basically no empirical support. It is plain to see that MO has a higher AIC value than any of the other models, where the difference is always greater than 10 except in the case of the last model.

**Table 1:** **Comparison of seven regression models**.

	***k***	**LL**	**AIC**	**ΔAIC**	**χ^2^**	***R*^2^**
MO	3	202.39	−398.77	48.06		0.83
MOA_*P*_B_*P*_	5	222.64	−435.27	11.55	40.50^***^	0.85
MOA_*G1*_B_*G1*_	5	216.72	−423.43	23.40	28.66^***^	0.85
MOA_*G2*_B_*G2*_	5	211.27	−412.53	34.29	17.76^**^	0.84
MOA_*G*3_B_*G*3_	5	228.41	−446.83	0.00	52.06^***^	0.86
MOA_*SS*_B_*SS*_	5	208.27	−406.53	40.30	11.76^*^	0.84

**k* is the number of parameters and LL the log-likelihood of each model. AIC is the Akaike Information Criterion, an index for model selection that takes model fit (i.e., log-likelihood) and model complexity (i.e., number of parameters) into account. ΔAIC is the AIC value minus the smallest AIC value. Models with smaller indices provide a more parsimonious (i.e., better) description of the data. *R*_2_ is the squared correlation between the fitted and observed values*.

* *p < 0.05*,

** *p < 0.01*,

*** *p < 0.001*.

Furthermore, we see that it makes a large difference which measure is used to calculate degrees of explanatory goodness. In particular, the model which includes next to O also A_*G*3_ and B_*G*3_ as predictors—so degrees of explanatory goodness obtained via *L*_2_—does best: it has the lowest AIC value of all models, the difference each time being greater than 10, and it has the highest *R*_2_ value (although in this respect all models are close to each other). This is confirmed by applying closeness tests for non-nested models to pairs of models consisting of MOA_*G*3_B_*G*3_ and one of the other explanationist models. Using Vuong's (1989) model, MOA_*G*3_B_*G*3_ is significantly preferred over any of the other explanationist models (in each case, *p* < 0.01), except for MOA_*G1*_B_*G1*_; in a comparison of MOA_*G*3_B_*G*3_ with MOA_*G1*_B_*G1*_, Vuong's test has no preference for either model. On the other hand, using Clarke's (2007) test, we find that MOA_*G*3_B_*G*3_ is preferred over *all* other explanationist models (in each case, *p* < 0.0001). Table 2 gives the regression results for MOA_*G*3_B_*G*3_. That O, A_*G*3_, and B_*G*3_ are all highly significant buttresses Douven and Schupbach's (in press) suggestion that when people receive new evidence, they change their credences not only on the basis of objective probabilistic considerations, but also on the basis of explanatory considerations. (At least, it supports that claim in the light of Schupbach's (2011) findings, which indicate a close match between subjective judgments of explanatory goodness and degrees of explanatoriness as calculated by any of the measures at issue.)

**Table 2:** **Regression results for the best explanationist model MOA_*G*3_B_*G*3_**.

**Variable**	***B***	***SE B***	**β**	***t***	***p***
Intercept	0.33	0.02		14.90	<0.0001
O	0.40	0.04	0.56	9.72	<0.0001
A_*G*3_	0.24	0.03	0.30	7.48	<0.0001
B_*G*3_	−0.13	0.03	−0.15	−3.67	0.0002

Finally, it is worthwhile comparing MOA_*G*3_B_*G*3_ (the best model with degrees of explanatory goodness determined via *L*_2_) with MOAB [the best model from Douven and Schupbach (in press) incorporating recorded judgments of explanatory goodness]. As previously remarked, the *R*_2_ value of MOAB equals 0.90. Its AIC value equals −519.64. So, on both counts, MOAB does better. MOAB is also preferred over MOA_*G*3_B_*G*3_ according to Vuong's test (*p* < 0.001) as well as according to Clarke's test (*p* < 0.0001). This implies that, *if* judgments of explanatory goodness are at hand, then one does best to take them into account in predicting people's updates. As noted, however, very often one will not have a choice, inasmuch as judgments of explanatory goodness are typically unavailable.

In fact, if judgments of explanatory goodness are available, one can even consider constructing a model that includes *both* variables encoding those judgments *and* variables encoding degrees of explanatory goodness, for instance, based on *L*_2_. Doing this for the present case, we find that in a model with all of O, A, B, A_*G*3_, and B_*G*3_, as predictors, B_*G*3_ is no longer significant. However, the model with the remaining variables as predictors does significantly better than MOAB in a likelihood ratio test: χ^2^_(1)_ = 9.12, *p* = 0.003. Also, the expanded model has a lower AIC value: −526.76. The *R*_2_ value is the same (0.90) for both models.

Summing up, we have found evidence that, at least in some contexts, explanationism is descriptively superior to Bayesianism: by taking explanatory considerations into account, next to conditional probabilities, we arrive at more accurate predictions of people's updates than we would on the basis of the objective conditional probabilities alone. Naturally, the kind of context we considered is rather special, and more work is needed to see how far the results generalize. Nonetheless, our results weigh against the generality of the increasingly popular hypothesis that people tend to update by means of Bayes's Rule.

## 4. Explanationism vs. bayesianism: normative adequacy

Here is a natural response to the findings of the previous section: “Surely people's updates do indeed break with Bayes's Rule. But this is unsurprising. Bayes's Rule is best interpreted as a norm of proper or rational updating in the light of new evidence. It is an idealization that actual agents can at best hope to approximate, to the extent that they are reasoning as they should. Even if experimental evidence calls descriptive Bayesianism into question then, it does nothing to invalidate Bayesianism as an ideal, normative theory.” In this section, we challenge this idea, summarizing recent work that compares Bayes's Rule with explanationist models of updating in order to clarify their respective roles in a full normative theory of rational updating.

Consider the so-called dynamic Dutch Book argument, which has convinced many philosophers that Bayes's Rule is the only rational update rule^3^. This argument has concomitantly done much to discredit explanationism as a normative account. The argument proceeds by describing a collection of bets, some of which are offered to a non-Bayesian updater before that person's update on new information and some of which are offered to him or her after that event. The claim is that, whatever the specifics of the update rule used by the person (other than that it deviates from Bayes's Rule), the pay-offs of the bets can be so chosen that all of them will appear fair in the eyes of the updater at the moment they are offered, yet jointly they ensure a negative net pay-off (such a collection of bets is called “a dynamic Dutch book”). This betokens irrationality on the updater's part—it is claimed—given that the updater could have seen the loss coming. Conversely, it is argued that had the person updated via Bayes's Rule, he or she could not have deemed all bets in the dynamic Dutch book to be fair.

There are at least three reasons for being dissatisfied with this argument. First, Douven (1999) points out that, in the dynamic Dutch book argument, what makes the non-Bayesian updater vulnerable to a dynamic Dutch book is not the use of a non-Bayesian update rule *per se*, but rather the combination of that rule and certain decision-theoretic principles, notably ones for determining the fairness of bets. As argued in the same paper, update rules must be assessed not in isolation, but as parts of *packages* of rules, which include decision-theoretic rules and possibly further update rules. Making use of a decision-theoretic principle proposed in Maher (1992), Douven demonstrates the existence of packages of rules that include a non-Bayesian update rule but that nevertheless do not leave one susceptible to dynamic Dutch books.

Second, even if non-Bayesian updating did make one vulnerable to dynamic Dutch books, it would not follow that such updating is necessarily irrational. For the possibility has *not* been ruled out that non-Bayesian updating has advantages that outweigh any risk of suffering financial losses at the hands of a Dutch bookie. It has recently been shown, in the context of a coin-tossing model in which it is unknown whether the coin is biased and if so what bias it has, that by updating via probabilistic abduction, one is on average faster—virtually always *much* faster—in attributing a high probability (explicated as a probability above 0.09, for instance) to the true bias hypotheses than if one updates via Bayes's Rule (Douven, 2013). Various philosophers have argued that high probability is a necessary condition for rational assertion and action: to be warranted in asserting or acting upon a proposition, the proposition must be highly probable. What this means is that a non-Bayesian scientist may get in a position to assert (including publish) the outcomes of his or her research more quickly than a Bayesian scientist who is working on the same theoretical problems. Or a non-Bayesian stock trader may be sooner warranted in making a profitable buy or sell than the Bayesians on the floor are, simply because he or she is quicker in assigning a high probability to the hypothesis that a given firm is going to do very well (or very poorly). Hence, for all Bayesians have shown, even if non-Bayesian updater's expose themselves to Dutch bookies, the financial losses they thereby risk incurring may be more than compensated for in other ways—*inter alia*, non-Bayesians's credences may converge toward the truth more quickly than those of their Bayesian competitors.

Third, even many Bayesians have become dissatisfied with the dynamic Dutch book argument. Above, it was said that the argument heavily depends also on what decision-theoretic principles are assumed. However, such principles would seem out of place in debates about *epistemic* rationality, which concern what it is rational to *believe*, or how to rationally change one's *beliefs* or *credences*, and not how it is rational to *act*. When we talk about rational action (e.g., the rationality of buying a bet), the notion of rationality at play is that of *practical* or *prudential* rationality. Even if Bayesian updating were the rational thing to do, practically speaking, it would not follow that it is the rational thing to do, epistemically speaking.

Motivated by this concern, Bayesians have sought to give an altogether different type of defense of their update rule. The alternative approach starts from the idea that update rules, like epistemic principles in general, are to be judged in light of their conduciveness to our epistemic goal(s), and that it is epistemically rational to adopt the update rule that is most likely to help us achieve our epistemic goal(s). The defense adopts inaccuracy minimization as the preeminent epistemic goal; update rules are accordingly epistemically defensible to the extent that they allow us to minimize the inaccuracy of our credences—where inaccuracy is spelled out in terms of some standard scoring rule(s). And according to Bayesians, it is their favored update rule that does best in this regard^4^.

It has recently been noted, however, that the goal of inaccuracy minimization, as it is used in the previous defense, is multiply ambiguous (Douven, 2013). That one ought to minimize the inaccuracy of one's credences can be interpreted as meaning that every update ought to minimize *expected* inaccuracy, but also as meaning that every update ought to minimize *actual* inaccuracy, or again differently, that every update ought to contribute to the long-term project of coming to have a minimally inaccurate representation of the world. And if understood in the third sense, there is the further question of whether we should aim to have minimally inaccurate degrees of belief in the long run, irrespectively of how long the run may be, or whether we should aim at some reasonable trade-off between speed of convergence and precision (see Douven, 2010).

What has effectively been shown is that Bayes's Rule minimizes inaccuracy in the first sense. However, no argument has been provided for holding that minimizing inaccuracy in that sense trumps minimizing inaccuracy in one of the other senses. So, in light of results showing that, given these other interpretations of our epistemic goal, certain versions of abduction outperform Bayes's Rule in achieving that goal (Douven, 2013; Douven and Wenmackers, in press), the inaccuracy minimization defense fails.

The upshot is that there is currently no good reason to hold that Bayesianism describes the unequivocally superior normative theory of updating. Both arguments that implore us to believe otherwise—the dynamic Dutch book argument and the inaccuracy minimization argument—fail in this regard. Bayes's Rule may be the uniquely best at enabling us to achieve one particular epistemic goal (minimizing expected inaccuracy in the long run). But there are other epistemic goals that we might have, which also involve the minimization of inaccuracy and which seem equally legitimate. Relative to some of these, abduction proves to be more conducive than Bayes's Rule. Results reported in Douven (2013) suggest that the precise epistemic goal(s) we should seek to satisfy is a matter that depends on context. That would mean that in some contexts Bayes's Rule is the preferred choice while in others it is abduction. But that is enough reason to reject the idea that abduction is an aberrant update rule, generally inferior to Bayes's Rule.

## 5. Conclusion

Nothing that we have said here calls into question the value of the probabilistic turn in recent cognitive science. We do, however, take issue with the narrowness of the focus of work in this vein. While we think that there is much fruit to be gleaned from modeling (actual and ideal) credences using probabilities, doing so does not necessitate using a Bayesian account. We have strived here to exemplify a promising way to expand fruitful research being pursued in cognitive science and philosophy today: namely, by exploring the probabilistic terrain outside of Bayesianism.

Doing so, we found strong support for explanationism, both as a descriptive and normative theory. At least in certain contexts, people do seem to base their updates partly on explanatory considerations; and at least with respect to certain plausible epistemic ends, that is what they ought to do. The present Research Topic (in which this article has been placed) centers around the question of how to improve Bayesian reasoning. This question could be taken to presuppose that Bayesianism is the one apt model of uncertain reasoning, and that all departures from Bayesianism are in need of improvement, repair, or explaining-away. In the above, we have challenged these presuppositions. Our findings suggest that when people update their credences partly on the basis of explanatory considerations and thereby flout Bayesian standards of reasoning, that can be because doing so puts them in a better position to achieve their epistemic goals. So, at least in some contexts, we can improve *upon* Bayesianism by taking into account the explanatory merits or demerits of the objects of our credences. To put the message in different terms, instead of asking how to motivate people to reason more in accordance with Bayesian standards, we should ask whether making people more Bayesian is a good idea to begin with.

We suspect that the answer to this question will depend sensitively on context and on the specific epistemic goals that are most salient for an epistemic agent. More research is thus needed to explore when exactly people are non-Bayesians and when exactly they should be. Specifically, do people tend to rely on some version of abduction mostly in those contexts in which it is best for them to do so, and similarly for Bayes's Rule? Bradley (2005, p. 362) argues that Bayes's Rule “should not be thought of as a universal and mechanical rule of updating, but as a technique to be applied in the right circumstances, as a tool in what Jeffrey terms the ‘art of judgment’.” Indeed, a key element in the art of judgment may be the ability to judge when to rely on Bayes's Rule and when to rely on abduction or other rules. In addition to this, it may comprise the art of judging explanatory goodness, which also means: not perceiving explanations where there are none. As with every art, one would expect some people to be better at this than others. (As an anonymous referee rightly noted, conspiracy theorists are inclined to see explanations everywhere, and abductive reasoning is likely to hamper rather than help such people to achieve their epistemic goals.)

While the above is not a call to abandon Bayes's Rule across the board—in some contexts, it may be exactly the right rule to follow—our present findings do go straight against Bayesianism as philosophers commonly understand that position, namely, as the position that any deviance from Bayesian updating betokens irrationality. It is to be emphasized, however, that there is no apparent incompatibility between our findings and much of the work in psychology that commonly goes under the banner of Bayesianism. There is nothing in the writings of Chater, Evans, Oaksford, Over, or most of the other researchers commonly associated with the Bayesian paradigm in psychology that obviously commits them either to Bayes's Rule as a universal normative principle or to the hypothesis that, as a matter of fact, people generally do obey the rule^5^. Oaksford and Chater (2013, p. 374) are quite explicit in this regard when they end their discussion of belief change in the context of the new Bayesian paradigm in psychology with the remark that “it is unclear what are the rational probabilistic constraints on dynamic inference.” We hope to have shed some new light on this matter by showing that, at least in some contexts, we do well to heed explanatory considerations, both as epistemic agents and as researchers trying to predict the cognitive behavior of others. More generally, we hope to inspire further research on the descriptive and normative merits of probabilistic, but non-Bayesian accounts of human reasoning.

### Conflict of interest statement

The authors declare that the research was conducted in the absence of any commercial or financial relationships that could be construed as a potential conflict of interest.

## Footnotes

^1^In this paper, we use “update” in the general sense of belief change. It is worth noting that some authors in the Bayesian camp (e.g., Walliser and Zwirn, 2002; Baratgin and Politzer, 2011) use the term to designate a *particular type* of belief change.

^2^This is not to deny that there is also evidence in support of the descriptive adequacy of Bayesianism. See in particular Griffiths and Tenenbaum (2006); Tenenbaum et al. (2006); Gopnik and Tenenbaum (2007), and Oaksford and Chater (2007).

^3^The dynamic Dutch book argument was first published by Teller (1973), who attributed it to David Lewis. Lewis's handout containing the argument was later published (Lewis, 1999).

^4^See Rosenkrantz (1992) for an influential early attempt along these lines; it also contains a detailed exposition of scoring rules.

^5^While Bayes's Rule has a very central place in the work of Griffiths, Tenenbaum, and their collaborators (see, e.g., Griffiths and Tenenbaum, 2006; Tenenbaum et al., 2006), even these authors do not commit to the claim that Bayes's Rule is the only rational update rule, or the rule that people everywhere and always use to accommodate new information.

## References

[B1] Achinstein P. (2001). The Book of Evidence. Oxford: Oxford University Press.

[B2] Adler J. (1994). Testimony, trust, knowing. J. Philos. 91, 264–275 10.2307/2940754

[B3] Bach K. Harnish R. (1979). Linguistic Communication and Speech Acts. Cambridge, MA: MIT Press.

[B4] Baratgin J. Over D. E. Politzer G. (2013). Uncertainty and the de finetti tables. Think. Reason. 19, 308–328 10.1080/13546783.2013.809018

[B5] Baratgin J. Politzer G. (2011). Updating: a psychologically basic situation of probability revision. Think. Reason. 16, 253–287 10.1080/13546783.2010.519564

[B6] Bonawitz E. B. Lombrozo T. (2012). Occam's rattle: children's use of simplicity and probability to constrain inference. Dev. Psychol. 48, 1156–1164. 10.1037/a0026471 22201450

[B7] Boyd R. (1984). The current status of scientific realism in Scientific Realism, ed Leplin J. (Berkeley, CA: University of California Press), 41–82.

[B8] Bradley R. (2005). Radical probabilism and Bayesian conditioning. Philos. Sci. 72, 342–364 10.1086/432427

[B9] Burnham K. P. Anderson D. R. (2002). Model Selection and Multi-model Inference: A Practical Information-Theoretic Approach. Berlin: Springer.

[B10] Clarke K. (2007). A simple distribution-free test for nonnested hypotheses. Polit. Anal. 15, 347–363 10.1093/pan/mpm004

[B11] Douven I. (1999). Inference to the best explanation made coherent. Philos. Sci. 66, S424–S435 10.1086/392743

[B13] Douven I. (2010). Simulating peer disagreements. Stud. Hist. Philos. Sci. 41, 148–157 10.1016/j.shpsa.2010.03.010

[B14] Douven I. (2011). Abduction, in Stanford Encyclopedia of Philosophy, ed Zalta E. (Spring 2011). Available online at: http://plato.stanford.edu/entries/abduction/

[B15] Douven I. (2013). Inference to the best explanation, Dutch books, and inaccuracy minimisation. Philos. Q. 69, 428–444 10.1111/1467-9213.12032

[B16] Douven I. Schupbach J. N. (in press). The role of explanatory considerations in updating. Cognition. 10.1016/j.cognition.2015.04.017 26069937

[B17] Douven I. Verbrugge S. (2010). The Adams family. Cognition 117, 302–318. 10.1016/j.cognition.2010.08.015 20961534

[B18] Douven I. Wenmackers S. (in press). Inference to the best explanation versus Bayes' rule in a social setting. Br. J. Philos. Sci.

[B19] Doya K. Ishii S. Pouget A. Rao R. P. N. (2006). Bayesian Brain. Cambridge, MA: MIT Press.

[B20] Elqayam S. Evans J. B. T. St. (2013). Rationality in the new paradigm: strict versus soft Bayesian approaches. Think. Reason. 19, 453–470 10.1080/13546783.2013.834268

[B21] Evans J. B. T. St. (1982). The Psychology of Deductive Reasoning. London: Routledge.

[B22] Evans J. B. T. St. Over D. E. (1996). Rationality and Reasoning. Hove: Psychology Press.

[B22a] Evans J. B. T. St. Over D. E. (2004). If. Oxford: Oxford University Press.

[B23] Fricker E. (1994). Against gullibility, in Knowing from Words, eds Matilal B. K. Chakrabarti >A. (Dordrecht: Kluwer), 125–161.

[B24] Friston K. J. Stephan K. E. (2007). Free-energy and the brain. Synthese 159, 417–458. 10.1007/s11229-007-9237-y 19325932 PMC2660582

[B25] Gigerenzer G. Hoffrage U. (1995). How to improve Bayesian reasoning without instruction: frequency formats Psychol. Rev. 102, 684–704 10.1037/0033-295X.102.4.684

[B26] Good I. J. (1960). Weight of evidence, corroboration, explanatory power, information and the utility of experiment. J. R. Stat. Soc. B22, 319–331.

[B27] Gopnik A. Tenenbaum J. B. (2007). Bayesian networks, Bayesian learning and cognitive development. Dev. Sci. 10, 281–287. 10.1111/j.1467-7687.2007.00584.x 17444969

[B28] Griffiths T. L. Tenenbaum J. B. (2006). Optimal predictions in everyday cognition. Psychol. Sci. 17, 767–773. 10.1111/j.1467-9280.2006.01780.x 16984293

[B29] Harman G. (1965). The inference to the best explanation. Philos. Rev. 74, 88–95 10.2307/2183532

[B30] Heit E. (2007). What is induction and why study it?, in Inductive Reasoning, eds Feeney A. Heit E. (Cambridge: Cambridge University Press), 1–24.

[B31] Heit E. Feeney A. (2005). Relations between premise similarity and inductive strength. Psychon. Bull. Rev. 12, 340–344. 10.3758/BF03196382 16082816

[B32] Heit E. Rotello C. M. (2010). Relations between inductive reasoning and deductive reasoning. J. Exp. Psychol. Learn. Mem. Cogn. 36, 805–812. 10.1037/a0018784 20438276

[B33] Hobbs J. R. (2004). Abduction in natural language understanding, in The Handbook of Pragmatics, eds Horn L. Ward G. (Oxford: Blackwell), 724–741.

[B34] Hohwy J. (2013). The Predictive Mind. Oxford: Oxford University Press.

[B35] Jeffrey R. (2004). Subjective Probability: The Real Thing. Cambridge: Cambridge University Press.

[B36] Josephson J. R. Josephson S. G. (eds.). (1994). Abductive Inference. Cambridge: Cambridge University Press.

[B37] Joyce J. (2009). Accuracy and coherence: prospects for an alethic epistemology of partial belief, in Degrees of Belief, eds Huber F. Shmidt-Petri C. (Dordrecht: Springer), 263–300.

[B38] Koehler D. J. (1991). Explanation, imagination, and confidence in judgment. Psychol. Bull. 110, 499–519. 10.1037/0033-2909.110.3.499 1758920

[B39] Legare C. H. Lombrozo T. (2014). Selective effects of explanation on learning in early childhood. J. Exp. Child Psychol. 126, 198–212. 10.1016/j.jecp.2014.03.001 24945685

[B40] Lewis D. (1999). Why conditionalize?, in Papers on Metaphysics and Epistemology, ed Lewis D. (Cambridge: Cambridge University Press), 403–407.

[B42] Lipton P. (1993). Is the best good enough? Proc. Aristotelian Soc. 93, 89–104. 15127986

[B43] Lipton P. (2004). Inference to the Best Explanation, 2nd Edn. London: Routledge.

[B44] Lombrozo T. (2006). The structure and function of explanations. Trends Cogn. Sci. 10, 464–470. 10.1016/j.tics.2006.08.004 16942895

[B46] Lombrozo T. (2007). Simplicity and probability in causal explanation. Cogn. Psychol. 55, 232–257. 10.1016/j.cogpsych.2006.09.006 17097080

[B47] Lombrozo T. (2012). Explanation and abductive inference, in Oxford Handbook of Think. Reason, eds Holyoak K. J. Morrison R. G. (Oxford: Oxford University Press), 260–276.

[B48] Lombrozo T. Carey S. (2006). Functional explanation and the function of explanation. Cognition 99, 167–204. 10.1016/j.cognition.2004.12.009 15939416

[B49] Lombrozo T. Gwynne N. Z. (2014). Explanation and inference: mechanical and functional explanations guide property generalization. Front. Neurosci. 8:700. 10.3389/fnhum.2014.00700 25309384 PMC4160962

[B49a] Maher P. (1992). Diachronic rationality. Philos. Sci. 59, 120–141.

[B50] Maher P. (1993) Betting on Theories. Cambridge: Cambridge University Press.

[B51] Mayo D. G. (1996). Error and the Growth of Experimental Knowledge. Chicago, IL: University of Chicago Press.

[B52] McMullin E. (1984). A case for scientific realism in Scientific Realism, ed Leplin J. (Berkeley, CA: University of California Press), 8–40.

[B53] McMullin E. (1992). The Inference that Makes Science. Milwaukee, WI: Marquette University Press.

[B54] Musgrave A. (1988). The ultimate argument for scientific realism in Relativism and Realism in Science, ed Nola R. (Dordrecht: Kluwer), 229–252.

[B56] Oaksford M. Chater N. (2007). Bayesian Rationality. Oxford: Oxford University Press.

[B57] Oaksford M. Chater N. (2013). Dynamic inference and everyday conditional reasoning in the new paradigm. Think. Reason. 19, 346–379 10.1080/13546783.2013.808163

[B58] Over D. E. (2009). New paradigm psychology of reasoning. Think. Reason. 15, 431–438 10.1080/13546780903266188

[B60] Pennington N. Hastie R. (1992). Explaining the evidence: tests of the story-model for juror decision making. J. Pers. Soc. Psychol. 62, 189–206 10.1037/0022-3514.62.2.189

[B61] Phillips L. D. Edwards W. (1966). Conservatism in a simple probability inference task. J. Exp. Psychol. 72, 346–354. 10.1037/h0023653 5968681

[B62] Popper K. R. (1959). The Logic of Scientific Discovery. London: Hutchinson 10.1037/0096-1523.11.4.443

[B63] Psillos S. (1999). Scientific Realism: How Science Tracks Truth. London: Routledge.

[B64] Psillos S. (2004). Inference to the best explanation and bayesianism, in Induction and Deduction in the Sciences, ed Stadler F. (Dordrecht: Kluwer), 83–91.

[B65] Rips L. J. (2001). Two kinds of reasoning. Psychol. Sci. 12, 129–134. 10.1111/1467-9280.00322 11340921

[B66] Robinson L. B. Hastie R. (1985). Revision of beliefs when a hypothesis is eliminated from consideration. J. Exp. Psychol. Hum. Percept. Perform. 11, 443–456.

[B67] Rosenkrantz R. D. (1992). The justification of induction. Philos. Sci. 59, 527–539 10.1086/289693

[B69] Schupbach J. N. (2011). Comparing probabilistic measures of explanatory power. Philos. Sci. 78, 813–829 10.1086/662278

[B70] Schupbach J. N. Sprenger J. (2011). The logic of explanatory power. Philos. Sci. 78, 105–127 10.1086/658111

[B71] Shalkowski S. (2010). IBE, GMR, and metaphysical projects, in Modality: Metaphysics, Logic, and Epistemology, eds Hale B. Hoffmann A. (Oxford: Oxford University Press), 167–187.

[B72] Thagard P. (2000). How Scientists Explain Disease. Princeton, NJ: Princeton University Press.

[B73] Teller P. (1973). Conditionalization and observation. Synthese 26, 218–258 10.1007/BF00873264

[B74] Tenenbaum J. B. Griffiths T. L. Kemp C. (2006). Theory-based Bayesian models of inductive learning and reasoning. Trends Cogn. Sci. 10, 304–318. 10.1016/j.tics.2006.05.009 16797219

[B75] Vuong Q. H. (1989). Likelihood ratio tests for model selection and non-nested hypotheses. Econometrica 57, 307–333 10.2307/1912557

[B76] Walliser B. Zwirn D. (2002). Can Bayes' rule be justified by cognitive rationality principles? Theory Decis. 53, 95–135 10.1023/A:1021227106744

[B77] Westfall R. S. (1980). Never at Rest. Cambridge: Cambridge University Press.

[B78] Williamson J. (2010). In Defence of Objective Bayesianism. Oxford: Oxford University Press.

[B79] Zhao J. Crupi V. Tentori K. Fitelson B. Osherson D. (2012). Updating: learning versus supposing. Cognition 124, 373–378. 10.1016/j.cognition.2012.05.001 22717167

